# Internal medicine residents’ perceptions and experiences in palliative care: a qualitative study in the United Arab Emirates

**DOI:** 10.1186/s12904-022-00908-5

**Published:** 2022-02-02

**Authors:** Thana Harhara, Dana Abdul Hay, Dalal S. Almansoori, Halah Ibrahim

**Affiliations:** 1 Department of Medicine, Sheikh Khalifa Medical City, Abu Dhabi, United Arab Emirates; 2 Department of Medicine, Tawam Hospital, Al Ain, United Arab Emirates; 3 Khalifa University College of Medicine and Health Sciences, Abu Dhabi, United Arab Emirates

**Keywords:** Palliative care, End-of-life care, Medical residents, Graduate medical education, Communication, United Arab Emirates

## Abstract

**Background:**

Palliative medicine is a newly developing field in the United Arab Emirates (UAE). The purpose of this study was to gain a deeper understanding of the experiences of internal medicine residents providing end-of-life care to patients and their families, and how those experiences shape their learning needs.

**Method:**

Nine focus groups were conducted with internal medicine residents and recent graduates from two large academic health centers in the UAE between 2019 and 2020. Through an iterative process, data were collected and examined using constant comparison to identify themes and explore their relationships.

**Results:**

Fifty-two residents and graduates participated. Residents frequently care for terminally ill patients and their families, but lack confidence in their skills and request more structured education and training. Cultural and system related factors also impact palliative care education and patient care. Five main themes and associated subthemes were identified: (1) clinical management of palliative patients, (2) patient and family communication skills, (3) religion, (4) barriers to end-of-life education, and (5) emotional impact of managing dying patients.

**Conclusion:**

Our findings can help guide program development and curricular changes for internal medicine residents in the region. Structured education in end-of-life care, with a focus on fostering culturally sensitive communication skills and spirituality, can improve resident education and patient care. Clear and transparent policies at the institution level are necessary. Programs are also needed to assist residents in developing effective coping strategies and emotionally navigating experiences with patient death.

**Supplementary Information:**

The online version contains supplementary material available at 10.1186/s12904-022-00908-5.

## Background

In developed countries, palliative medicine has emerged as a model of care to alleviate pain and suffering and increase dignity at the end-of-life [1]. The World Health Organization defines palliative care (PC) as “an approach that improves the quality of life of patients and their families who are facing problems associated with life-threatening illness. It prevents and relieves suffering through the early identification, correct assessment and treatment of pain and other problems, whether physical, psychosocial or spiritual.” [2] Evidence suggests that early referral to PC improves patient and caregiver quality of life and reduces the burden on the healthcare system [1]. Worldwide, however, there is a dearth of PC trained specialists, and in many nations, PC services are insufficient to meet population needs [3]. Approximately 6% of PC services globally are located in Africa and Asia [4].

The United Arab Emirates (UAE) provides an opportunity to gain insight into the perspectives of physicians-in-training in developing countries on palliative medicine. Over the past two decades, the UAE has developed its healthcare and health education infrastructure, with state-of-the art hospital facilities and international affiliations, including the Cleveland Clinic, Imperial College of London, and the Mayo Clinic. Comprehensive cancer centers are in development across the emirates. Also, many of the country’s medical training programs have been restructured to meet international standards and have received certification from the Accreditation Council for Graduate Medical Education International [5]. The ramping up of healthcare and medical education programs has occurred in parallel with an aging society and increasing incidence of cancer in the UAE [6]. Yet, there has been limited development of specialized PC facilities [7]. As such, most PC services in the UAE are provided in an acute hospital setting by general physicians and medical trainees. Significant gaps in the teaching and assessment of PC in medical education programs have been documented worldwide [8, 9]. Multinational studies confirm that both trainees and practicing healthcare professionals lack the necessary core knowledge and skills to provide adequate end-of-life care [10–12]. To our knowledge, there are no published studies exploring the viewpoints of residents providing PC in the UAE. The purpose of this study is to gain a deeper understanding of the perspectives and experiences of internal medicine (IM) residents in the UAE in providing PC to patients and their families, and how their experiences shape their learning needs.

## Methods

### Theoretical perspective

The theoretical underpinning of this study is based on Lave and Wenger’s theory of situated learning, in which learning is a contextual process that is situated within an environment and culture [13]. In this regards, residents learn about death and dying and end-of-life communication within the hospital environment and the specific religious and cultural contexts of the UAE.

### Interview guide

The interview guide was developed by TH, a clinician educator with training in palliative medicine, and was reviewed for length and clarity by three recent board certified graduates of the program, who did not participate in the final focus groups. Questions aimed to explore residents’ understanding of palliative care, perceived learning gaps, and challenges faced in providing PC. We also sought to gain a deeper understanding of resident experiences with death and dying in the context of their training. Data collection and analysis occurred concurrently and led to iterative adjustments of the interview guide.

### Setting and participants

Participants were internal medicine residents, years one through four, and recent graduates from two large academic medical centers in the UAE. These hospitals are tertiary care centers and oncology referral centers for the region. They sponsor the largest IM training programs in the country. Most of the residents graduated from medical schools in the Middle East. The residency program coordinator arranged the meeting times and sent email invitations to all residents. All respondents provided informed consent. Participation was voluntary. We grouped residents by year level to facilitate openness and minimize social desirability bias that may occur when junior and senior residents are grouped together.

### Data collection

From March 2019 through February 2020, we conducted nine focus groups - seven with IM residents and two with recent graduates. All of the interviews were conducted by one of the investigators (HI), who has experience with qualitative methodology and was not involved in administration or leadership of the residency program at the time. Each focus group had approximately five to seven participants and lasted 50 to 80 min. The focus group interviews were conducted in English, audio recorded, transcribed verbatim by professional transcribers and checked for accuracy and completeness. They were de-identified prior to data analysis, except for resident year level and gender. We stopped recruitment once no new themes emerged.

### Data analysis

All data analysis was manually performed by two of the investigators (HI and TH). We performed a qualitative analysis using a thematic content approach [14]. We independently performed line-by-line coding on the transcripts and generated initial codes. Through thematic analysis, we identified recurring concepts in the coded data relating to resident PC education and training, and then categorized them through constant comparison into themes [15]. All of the authors compared and discussed each theme until we reached consensus on a coding scheme, which was then applied to all the transcripts. We used the Standards for Reporting Qualitative Research (SRQR) checklist when writing our report [16]. Trustworthiness of results was reached by interviewing two groups (residents and graduates) and including two different hospitals, as well as by having two of the authors participate in the iterative data analysis. The Sheikh Khalifa Medical City institutional review board in Abu Dhabi, UAE approved the study [RS-564].

### Team reflexivity

We were mindful of how our backgrounds and experiences in resident education in the UAE influenced our analysis. Including a palliative care specialist (TH) and non-specialist physicians (DAM and HI), as well as a graduate of the residency program (DAH), brought diversity of perspective to the data analysis process with the intent of minimizing potential researcher bias.

## Results

A total of 52 individuals participated in the focus groups - 40 women and 12 men. Ages ranged from 25 to 34 years, with mean age of 27.7 years. Participants were evenly distributed amongst the year groups (12 year one, 12 year two, 10 year three, 11 year four, and 7 graduates). Five main themes and associated subthemes related to resident education were identified: (1) clinical management of PC patients, (2) patient and family communication skills, (3) religion, (4) barriers to end-of-life care education, and (5) emotional impact of managing dying patients. Each theme is discussed below and illustrated in Table 1 (Supplementary material), with excerpts from the resident discussions to evidence our findings. Of note, themes were consistent across year groups, and did not vary by hospital or gender.

### Theme 1: clinical management of PC patients

Most residents reported receiving limited formal education in PC symptom management and lacked confidence in their skills. Without formal training, learning occurred through observation of their seniors and faculty. One resident noted:

“We had some lectures on palliative care, but it wasn’t a full course. From our experiences with patients with advanced diseases in this hospital, we have some hints about it, but it’s not like a structured program.” (2^nd^ year female resident)

Trainees also found it difficult to shift from aggressive disease treatment to prioritizing symptom alleviation and quality of life. They expressed uncertainty about treatment decisions. One senior resident reflected:

“I think we are not doing the right thing. We are trying to save them, prolong their life, but the quality is not what it should be. Because what we do is more acute medicine most of the time, unless we have someone who will guide us … .But on the acute wards, we treat them as acute medicine, which I don’t think is the right thing for palliative patients.” (4^th^ year male resident)

### Theme 2: patient and family communication skills

The vast majority of residents requested additional training in end-of-life communication skills, and they were particularly interested in education to improve initiating goals of care and code status conversations. One graduate summed up her training experience:

“We definitely need more counseling and communication sessions … I never formally learned the right way to communicate with the patients, to choose certain words that are less alarming, words that would have been beneficial. But I learned through experience.” (female graduate)

Residents recognized that the family unit played a major role in healthcare decision making in the UAE, and that the concept of patient autonomy was primarily a Western construct. Yet, they struggled when tensions arose between these dynamics. A senior resident explained:

“I know relatives see things from a different perspective than physicians, but it’s very hard when I see them trying to ease their own guilt, rather than doing what’s best for their loved one. They don’t want to blame themselves for withholding something from the patient. It’s very hard to explain to them that these things are more harm than good.” (4^th^ year male resident)

### Theme 3: religion

The residents acknowledged that seriously ill patients and their families have religious and spiritual needs, but they felt unprepared to address these needs. One resident remarked:

“I usually don’t discuss religion unless the family asks. It’s not my job to advise. It’s not my field. So we usually try to avoid these conversations and tell them we’re just doing the medical part of it and religion is not our specialty.” (4^th^ year male resident)

Another resident recognized the deficiency in her response:

“I had a patient during the night, who had end stage cancer. We all knew that she was dying. Her brother approached me and asked me if DNR [do not resuscitate] is haram [a sin] in Islam. I didn’t know what to tell him. I spoke to him from a medical point of view. But until now, I don’t know the answer to his question. I think I should search in the Quran for an answer.” (1^st^ year female resident)

### Theme 4: barriers to end-of-life education

The residents perceived several barriers to their education in palliative medicine, namely cultural resistance from families, lack of knowledge or awareness of hospital guidelines and policies, and variation in the faculty’s medical practice. Residents also expressed several concerns regarding futility, ranging from uncertainty about the aggressiveness of their own management to distress over family decisions to continue care that they considered to be medically futile. One senior resident noted:

“I remember in my first year, the nurses called me for a palliative patient. The patient was hypoxic and I wanted to do an ABG [arterial blood gas] because that’s part of the work up. And I had to argue with the nurses because they were telling me that this patient is palliative … I didn’t have a good grasp on what is palliative care … How much should we do? When should we stop? What is aggressive care? Is inserting a cannula aggressive because it can cause pain? But I have to give fluids and IV antibiotics. This is the gray area.” (3^rd^ year female resident)

Trainees also felt that their uncertainty regarding hospital policies on end-of-life issues affected their care of seriously ill patients and caused greater frustration. For example, one resident asked:

“Seeing these patients suffering on a daily basis and me giving them more suffering, sometimes it makes me depressed because I feel like I’m not a good doctor. The patient wants some comfort and I’m not giving it to him. But I cannot break the rule here … We don’t have formal end-of-life policies like they do in foreign countries. It’s really difficult for me. I prefer not to have any palliative patients to take care of, because I feel like I’m confused about their management, what to treat, what not to treat?” (3^rd^ year female resident)

Another resident stated:

“I wish there was a protocol for advanced diseases, like we do a VTE [venous thromboembolism] form for each patient, but we don’t do a palliative care plan for them so that we know what to do.” (1^st^ year male resident)

Trainees also felt that the variation in practice among teaching faculty created confusion and uncertainty in managing terminal patients:

“That’s why the hospital needs protocols and proper education. Working with many different consultants [attendings], I’ve seen that they all have their own way of managing [palliative] patients … I see the discrepancies in care and it’s frustrating. Even for patients and families, they say one doctor did this, why aren’t you doing the same? And how do I respond? We should have a unified way of management.” (4^th^ year female resident)

### Theme 5: emotional impact of managing dying patients

All residents reported many emotional reactions when dealing with terminal patients, including guilt, sadness, and hopelessness. Feelings of failure were also expressed as deaths, even when expected, made the residents question their competence. One resident described the death of an elderly patient:

“She just deteriorated and died in less than a day. But it did make me feel very bad. And I felt very depressed. I felt very angry with myself, and I felt very doubtful of myself. For about two weeks, I was wondering how I’m going to manage other patients? I don’t know what happened with this patient? It was very horrible. Maybe I’m overthinking it. But it was really, really horrible for me.” (3^rd^ year female resident)

Both senior and junior residents agreed that the emotional impact of patient deaths was most challenging in the first year of training, and that trainees rarely sought or received support from faculty members. One resident explained:

“I didn’t speak to anybody because it was my first year. I didn’t know anybody, and I didn’t want to show that I was weak. I wanted to show that I was a good resident and that I can handle anything.” (2^nd^ year male resident)

Trainees described both effective and maladaptive coping mechanisms resulting from these interactions. One recent graduate admitted to avoiding palliative patients whenever possible:

“She was suffering in so much pain … So, everyday we had to watch her suffer. But we had to go in every day. You just don’t want to go in the room. If I’m on a team and it’s not my patient, I don’t go into these rooms.” (female graduate)

All trainees agreed that providing psychological support and training on effective coping strategies, primarily for junior residents, is necessary and worthwhile.

## Discussion

Internal medicine residents provide direct care to seriously ill and dying patients and their families, and are often expected to do so early in their training. Similar to other research, IM residents in our study reported deficiencies in PC education and lacked confidence in their skills [10–12]. Education programs to improve PC training and end-of-life symptom management, specifically including chronic pain management and symptom alleviation, should be requisite training in UAE medical schools and residency programs.

Communication in end-of-life care was highlighted by the trainees as a specific area of concern. Studies confirm that involving patients and their families in structured discussions about goals of care and end-of-life wishes can increase patient quality of life and improve family satisfaction with decision making [17]. As such, additional training in end-of-life clinical management and communication skills may not only increase resident competence and confidence, but also improve patient care and satisfaction.

In addition to their own insecurity or reluctance to initiate these conversations, residents discussed several cultural and systems issues that they perceived to be barriers to their education. The lack of clear policies regarding initiation of PC, do not resuscitate orders, and patient autonomy were cited as obstacles to patient care, as well as a source of emotional and moral distress. For example, residents admitted that they often provided “acute” and “aggressive” care to patients at the end-of-life, despite recognition that these treatments were likely non-beneficial. This finding is consistent with other studies [18]. Educational interventions, including rotations on palliative care wards or nursing homes, have been successful in educating trainees on when to initiate PC and improve symptom management skills [18], thereby improving the quality of care provided to terminal patients and their families.

Resident emotional distress from caring for terminal patients has been previously reported. A survey in Canada found that almost half of the resident respondents reported feelings of guilt or failure after a patient death [19]. Similarly, our residents revealed a myriad of negative emotions and frustrations regarding their interactions with palliative patients. Although residents did find support from peers and sought to develop effective coping strategies, several trainees described maladaptive coping mechanisms, including avoidance. Reported barriers to resident help-seeking behaviors include fear of stigma, concerns about privacy breaches, or worry that progression in the training program or later career trajectory might be negatively impacted [20]. Our residents echoed these concerns. There is a large body of literature linking physician distress with decreased empathy, impaired decision making and adverse patient outcomes [21, 22]. As such, programs that address resident wellbeing may also improve patient care outcomes. Research has shown that single point interventions, such as a workshop, a meeting with a psychologist or debriefing after a patient death, are insufficient to fully address resident concerns [23]. Instead, a multi-dimensional and holistic approach to resident wellbeing is essential, and should include training in personal reflection and building effective coping strategies. Incorporating faith and spirituality in developing resilience may be particularly helpful for trainees in the region. Future studies of these interventions are important to assess effectiveness.

Further, our trainees recognized the high prevalence of religious concerns amongst their seriously ill patient population. Data suggests that terminal patients who have unmet spiritual needs have significantly poorer quality of life [24]. However, studies show less than 50% of physicians feel that it is their role to address these needs [25]. In many countries, chaplains or other faith leaders fulfill this role [26]. As this position currently does not exist in UAE hospitals, it is important to inculcate religious and spiritual training into the PC curriculum for medical residents. Professional development programs focusing on helping patients find meaning, purpose and connectedness, would likely be helpful for all healthcare professionals in the region [27].

Our findings have several important implications for curricular and policy reform. Although specialized education programs in palliative medicine, including fellowship training, exist in several countries, formal training in PC does not currently exist in the UAE. Further, UAE medical schools and residency programs currently lack structured PC curricula [9]. Successful interventions for improving IM resident knowledge and comfort in PC have been reported, including mandatory rotations in PC and communication skills workshops [28, 29]. Systematic reviews have shown improvement in trainee skills and confidence, regardless of the educational method employed [29]. The vast majority of these studies, however, were conducted in countries where PC services are well established. Studies in developing regions are important to assess efficacy of various teaching and policy interventions in different cultural contexts. Also, improved physician knowledge of end-of-life policies in the hospitals is essential. The UAE established a national policy in 2016 to allow natural death in patients suffering from a terminal illness [30]. Many hospitals have already adopted these regulations. Awareness and education programs are necessary to familiarize all healthcare providers with current hospital protocols. Further, training in shared decision-making and cultural competence can help all physicians to better navigate issues regarding patient dynamics and patient-family tensions. More local research is needed on these important topics.

### Strengths and limitations

This study adds to the PC literature by providing the perspectives of medical residents in an emerging healthcare and medical education system. Strengths include the inclusion of trainees from the two largest IM residency programs in the country. Also, residents from all year levels participated in the focus groups. Ours study, however, has some limitations. First, our respondents were primarily women, though UAE residency programs consist of predominantly women trainees. Female gender predominance has been noted in other residency programs in the regions [31]. Also, the findings represent the residents’ perceived barriers to PC training. The perspectives of other stakeholders, including faculty and patients, should be included to comprehensively identify and address barriers to end-of-life care provision. Finally, contextual factors, including religion and culture potentially impacted the residents’ views regarding palliative and end-of-life care, though studies of physicians in non-Arab and Western countries have shown similar results.

## Conclusion

Proficiency in providing high quality palliative care is important for healthcare workers worldwide. This study has highlighted educational and policy issues faced by our IM residents. We believe our findings and recommendations are generalizable and can help other residency programs and academic health centers improve palliative care provision for patients and families in the region.

## Supplementary Information

**Additional file 1.**

## Data Availability

No quantitative datasets were generated during this study. The qualitative data generated are not publicly available to maintain participant and institution confidentiality but are available from the corresponding author upon reasonable request.

## Abbreviations

IM: Internal Medicine
PC: Palliative care
UAE: United Arab Emirates
