# Supplementary material for: Internal medicine residents’ perceptions and experiences in palliative care: a qualitative study in the United Arab Emirates
Source: BMC Palliat Care. 2022 Feb 2;21:15. doi: 10.1186/s12904-022-00908-5 (PMC8809043; doi:10.1186/s12904-022-00908-5)
Supplement: Supplementary file 1 — Additional file 1. [file 12904_2022_908_MOESM1_ESM.docx]

**Table 1. Internal Medicine Residents’ Experiences With Palliative Care Education: Themes and Supporting Quotes**

| **Themes** | **Subthemes** | **Quotes** |
| --- | --- | --- |
| 1. **Clinical management of palliative care patients** | | |
|  | Lack of structured training | We had a short presentation on end of life care, but it wasn’t enough detail to help me manage these issues. For example, I feel we need more information about pain management… But I don’t remember receiving any details about palliative care, what things to do, what things not to do. |
|  | Evolution in understanding of palliative care | You start to accept that this is part of the cycle of life. There isn’t much more that you can do. Once a patient reaches a palliative stage, then all you can do is emotionally support the patient. Be there for them and improve the quality of their life in whatever way you can. |
| 1. **Patient and family communication skills** | | |
|  | Patient autonomy vs. family unit | I saw a patient in the ICU rotation, who had a husband who had a trach [tracheostomy]. When she saw him suffer, she decided that she didn’t want any tubes. Despite this, when she deteriorated after many years, her family decided to trach her, but she didn’t want it in the first place. She ended up having a trach although it was against her will. I think her wishes should have been taken into consideration. |
|  | Learning goals of care conversations | When we started doing nights early on, we had to have these conversations… My discussions at the beginning were like do you want intubation or not, do you want ICU or not. But now I try and ask more about the patient’s wishes. Just because we’re more experienced with it, we bring them up during discussions. |
|  | Role modeling | I watched family meetings in the ICU. I would see the consultant talking to the family and watch how to open up the discussion, how to start answering their questions, how to help them choose what is right- just by giving them all the options and addressing everything and telling them about the consequences of an action that is needed to be taken. That is one way of learning. |
| 1. **Religion** | | |
|  |  | I think I was once asked this question when I was talking end-of-life with the family and they said, where does the soul go? Where is the soul right now? … I didn’t know what to answer to that, honestly. Where is the soul right now? That actually made me think about the soul. |
| 1. **Barriers to end-of-life care education** | | |
|  | Cultural resistance to palliative care | And some families get shocked when you discuss end-of-life care. They say we brought our family member to the hospital for you to take care of them- so they get shocked. I think our community is not fully aware of the limitations of medicine. |
|  | Institutional policies and legal implications | It depends on the hospital policy. We don’t have a formal end-of-life protocol here. That’s why we do extensive management. It’s difficult for me … But I cannot break the rule here. |
|  | Variations in medical practice | I work with different consultants [attending physicians] and I see different plans. Nothing is fixed. Maybe that’s why I can’t manage it on my own… Working as a junior, it always confused me. One consultant would say stop this, but the other consultant would say to continue it. So this confusion is why I cannot initiate the management on my own. |
| 1. **Emotional impact of managing dying patients** | | |
|  |  | It will stick with me as a learning experience [1^st^ patient death]. I wasn’t ready for it. I did not expect it. I thought it was so difficult. When the ICU came, they told me to ‘keep breathing, don’t cry, be strong in front of the family. It’s not appropriate for a doctor to act like this.’ I did not cry but I had watery eyes. I knew the patient well. And I knew the family, his daughters. That’s why I was so affected by it. |
